# Butterfly Density and Behaviour in Uncut Hay Meadow Strips: Behavioural Ecological Consequences of an Agri-Environmental Scheme

**DOI:** 10.1371/journal.pone.0134945

**Published:** 2015-08-18

**Authors:** Julie Lebeau, Renate A. Wesselingh, Hans Van Dyck

**Affiliations:** Biodiversity Research Centre, Earth and Life Institute, Université catholique de Louvain (UCL), Croix du Sud 4–5, box L7.07.04, B-1348, Louvain-la-Neuve, Belgium; Oxford Brookes University, UNITED KINGDOM

## Abstract

Sparing zones from mowing has been proposed, and applied, to improve local conditions for survival and reproduction of insects in hay meadows. However, little is known about the efficiency of refuge zones and the consequences for local populations. We studied population densities of butterflies before and after mowing in the refuge zone of 15 meadows in 2009 and 2011. We also studied the behaviour of the meadow brown (*Maniola jurtina*) comparing nectar use, interactions and flights in the refuge zone before and after mowing. Densities of grassland butterflies in this zone doubled on average after mowing. The density of females of *M*. *jurtina* increased on average fourfold, while males showed a more modest increase. In line with the idea of increased scramble competition in the refuge zone after mowing, *M*. *jurtina* increased the time spent on nectar feeding, the preferred nectar source was visited more frequently, and females made more use of non-preferred nectar sources. *Maniola jurtina* did not interact more with conspecifics after mowing, but interactions lasted longer. Flight tracks did not change in linearity, but were faster and shorter after mowing. After mowing, only a part of the local grassland butterflies moved to the uncut refuge zone. The resulting concentration effect alters the time allocated to different activities, nectar use and movements. These aspects have been largely ignored for agri-environmental schemes and grassland management in nature reserves and raise questions about optimal quantities and quality of uncut refuge sites for efficient conservation of grassland arthropods in agricultural landscapes.

## Introduction

Agriculture has strongly intensified since the 1950s with well-documented negative impacts on biodiversity [1,2]. Semi-natural grasslands under extensive management typically have species-rich communities [3], but their significance for agriculture has declined considerably; most permanent grasslands have been turned into intensively managed grasslands (with several cuts per year and selected species) or crop fields [4]. The role of remnants of semi-natural grassland is, however, also recognized in intensive, agricultural landscapes, as these habitats function as population sources of pollinators and other agro-biodiversity [5,6]. In hay meadows, agricultural intensification results in homogenization of the vegetation structure and decline in plant diversity [7]. This in turn affects animals that rely (in)directly upon plant resources and associated environmental conditions.

Extensive grassland management has become a conservation practice in protected areas to control ecological succession of open habitats. Although disturbance, like mowing, is essential to keep semi-natural grasslands from successional development towards shrubland, mowing regimes may also result in increased direct and indirect mortality of grassland fauna, especially invertebrates [8,9]. Direct mortality caused by the physical impact of mowing can be significant for relatively sedentary invertebrates[8], juvenile insect stages [10] and ground nesting birds [11]. For flying insects (e.g. butterflies), increased direct mortality may only occur when mowing is done at a time of day when these ectotherms show reduced activity [10,12]. However, the most significant impact of mowing is likely to be an indirect effect on resource availability through the sudden disappearance of consumables (especially floral nectar) and altered local microclimatic conditions [9,13]. Depending on the spatial extent and the timing of mowing, indirect effects may alter individual survival and population viability.

Mowing regimes that leave particular zones uncut have been recommended [10,12,14]. This body of work provides the rationale for particular agri-environmental schemes and also for rotational mowing management of grasslands in nature reserves [15]. To the best of our knowledge, there are surprisingly few studies that show the quantitative effects of uncut refuges in grasslands on specific organisms (but see[16]).

Here we address the practice of leaving refuge strips unmown in hay meadows under agri-environmental schemes focusing on butterflies. We tested the hypothesis of increased use of the uncut refuge zones after mowing. This is often assumed, but rarely tested. After mowing, the majority of the surface of a meadow is cleared from floral nectar resources. This may induce dispersal away from the meadow, but also a concentration effect in the refuge zone. The latter case may in turn alter the living conditions for butterflies (and other insects) in the refuge. Depending on the surface of the refuge and its resources relative to the consumer populations, this may provide scope for further indirect, (sub)lethal effects of mowing regimes. Potential mechanisms include increased competition for nectar [17], increased predation rates (e.g. [18]) and increased male harassment for females (e.g. [19]).

Butterflies are popular study species in the context of agri-environmental schemes (AES), but most studies on these schemes focus on agricultural fields rather than on grasslands [20–26]. AES-related studies typically monitor species abundance and diversity between sites submitted to AES and conventionally managed sites (e.g. [21,25,27]). Here we tested two main hypotheses. First, we tested for a concentration effect of grassland butterfly species [28] in the refuge after mowing contrary to other habitat generalist butterfly species, and more specifically, we tested for such a concentration effect in the grassland species *Maniola jurtina* L. Second, we tested to what extent butterfly life changed in refuge zones by comparing behaviour and resource use (frequencies of intraspecific interactions, changes in time budget for different activities and differences in foraging flight paths) before and after mowing in *M*. *jurtina*. Assuming increased scramble competition, we predicted that *M*. *jurtina* butterflies spended more time feeding after mowing. Rewards per flower visit were likely to be smaller, among-butterfly interactions more frequent and flower visits shorter on average. Under this scenario of increased scramble competition for the preferred nectar, *M*. *jurtina* was predicted to use less-preferred nectar plants more frequently in the refuge after mowing. Finally, we tested the prediction of altered flight pathways with shorter flight bouts in the refuge after mowing.

## Material and Methods

### Study sites and agri-environmental scheme

Study sites were located in a 10 x 10 km area in Belgium (municipalities: Houyet, Beauraing and Rochefort). The landscape mainly consists of forest and grassland, both pastures and hay meadows. We collected data in 15 different hay meadows of various size (range: 0.7–15 ha). All sites were under agri-environmental scheme agreement, which means that meadows cannot be mown before June 15. The sites were located on private lands, and owners gave permission to conduct the study on their lands. The agreement includes limited or no use of manure and bans the use of pesticides. Hay must be removed after mowing and at least 5–10% of the meadow should be left uncut. The location of the unmown refuge zone is allowed to vary between years. Generally, the unmown zone is located on the edge of the meadow and is long and narrow (typically 3 to 5 m large). During our study, all sites were mown, but not grazed.

### Study species

We recorded several species of specialist grassland butterflies and generalist butterflies, which may be found in meadows but are not confined to this habitat (as defined in [28]). The former group would find most of its resources (e.g. host plants) in the meadows. For all specific behavioural measures we focused on *Maniola jurtina*. This satyrine is a widespread grassland species, but recent data in the framework of the European grassland butterfly index suggest a decline at the European scale [29]. It is strictly univoltine and males appear up to 10 days before females [30]. In Belgium, adults are present from late May to the end of August. Adults live on average 5 to 12 days in the wild [30]. Caterpillars feed on a variety of *Poaceae*; adults visit several flower species [24,30]. Males spend more time flying than do females [31]. Males patrol to locate receptive females, or alternatively, perch on the vegetation to intercept passing females. Unlike other butterfly species, *M*. *jurtina* is not territorial, i.e. males do not defend a specific perching spot as in *Pararge aegeria* for instance [32]. Females alternate periods of resting with feeding and egg laying and feed more frequently than males [30]. The average individual of a population is not very dispersive and the typical action radius is 300 m or less, however, estimates depend on the scale of the study and movements have observed up to 1 km [33] and more (4700 m, Lebeau et al., unpublished data). Hence, the species is not considered as a sedentary species, but it is clearly not as mobile as other butterfly species that typically fly over several kilometres every day [34,35]. Our field study did not involve endangered or protected species.

### Butterfly density and activity

Butterfly numbers were counted in ten sites in 2009 (June 18–August 4) and in seven in 2011 (June 9–August 2), two of which were also studied in 2009, between 10.00 h and 17.00 h on sunny days (temperature > 18°C, no strong wind) on 5 m x 20 m transects (Pollard walk method; Pollard and Yates 1993). Each site was visited three times before and three times after mowing. The mean number of days (± SE) between the date of the transect count of the three visits and the date of mowing was 7.6 ± 0.6 days before mowing and 4.7 ± 0.7 days after.

Sites were chosen to reflect variation in mowing date relative to butterfly phenology. In 2009, the studied meadows were mown 2 weeks (end of June), 4 weeks (mid July) and 6 weeks (end of July) after emergence of *M*. *jurtina*. In 2011, meteorological conditions forced farmers to mow at the beginning of July, one month after the beginning of the flight period. Hence, the combined data set provides a reasonable level of overlap between mowing dates and the butterfly flight season without any particular phenological bias. Additionally, we performed independent transect counts throughout the study period in 2011 in one site that was never mown, to provide reference phenology curves without mowing. This reference site, located within the study area, was visited on 7 days and three transects were done at each date. Butterflies were recorded in the same categories (i.e. grassland specialist species or other habitat generalist species) as for mown sites [28].

Future refuge zones were delineated before the flight period and we did one transect count per visit per site in this zone before and after mowing. The abundance of all butterfly species was recorded and we distinguished between grassland (e.g. *Coenonympha pamphilus*, *Maniola jurtina*, *Aphantopus hyperantus*, *Melanargia galathea*, *Pyronia tithonus*, *Papilio machaon*, *Thymelicus lineola*, *T*. *sylvestris*, *Thecla betulae*, *Heodes tityrus*, *Polyommatus icarus* and *Lycaena phlaeas*) and habitat generalist species (e.g. *Aglais urticae*, *A*. *io*, *Arashnia levana*, *Vanessa cardui*, *V*. *atalanta*, *Aporia crataegi* and pierid butterflies) [28]. For *M*. *jurtina* we also recorded sex and activity (flying, resting/basking, mating, egg-laying or feeding).

Floral nectar supply was quantified in each meadow every 10 days. Along each transect, all flower units were counted by plant species in ten randomly placed 1-m^2^ plots. One inflorescence or one solitary flower equalled one flower unit. The sum of all flower units in all plots of each transect was used as an estimate of the total nectar supply. We also calculated the ratio of the abundance of *Centaurea jacea* L. relative to the abundance of other flower species. In our sites, *C*. *jacea* is the preferred nectar source of *M*. *jurtina* and of several other butterflies.

### Competition for nectar

In 2009, we introduced a number of potted nectar plants to one of our sites (Site CO; 50°07’54”N– 5°04’12”E) on sunny days between 10.00 h and 17.00 h and recorded flower head visits by *M*. *jurtina*. We used two nectar plant species: the preferred *C*. *jacea* and *Trifolium pratense* L. The latter is typically used in the absence of *C*. *jacea*. The individuals of both nectar species were grown in pots under outdoor conditions. Plants that were introduced in the field were clipped back to a single inflorescence, protected by an exclosure bag to avoid insect visits prior to the experiment. We placed one flowering plant of each species next to each other in the refuge zone. The exclosure bags were removed and flower visits were recorded during 1 h by video camera (JVC Everio). As we could not distinguish between males and females in every sequence, we used total visit rate by *M*. *jurtina*. We repeated the experiment on 5 days before mowing (between June 25 and July 16) and on 4 days after mowing (between August 1 and August 12).

### Behavioural tracking

In the same year and site, we tracked the behaviour of 41 males (28 before and 13 after mowing) and 68 females (39 before and 29 after mowing) on sunny days between 10.00 h and 17.00 h. Butterflies were caught with a hand net and individually marked on the ventral hind wing. After marking, the butterfly was returned to the net and was released after a 1-min recovery period. Next, the butterfly was tracked for 10 min from a distance of 1 m. The trajectory was recorded by a handheld GPS (Pathfinder Data logger SiRf Star III, GPS-DL R8; relative spatial accuracy: ± 0.1 m). The following behavioural categories were recorded using a digital voice recorder (Olympus VN-5500PC): flying, feeding, basking, resting, mating, egg laying (females) and intraspecific interactions (male-male and male-female), which were used as a measure of the intensity of scramble competition. For flower visits, the nectar plant species was also recorded. Before mowing, tracking was performed in the whole site, since no butterfly stayed within the future refuge zones, which did not differ from the rest of the meadow at that time and were smaller than an individual butterfly’s home range. After mowing, tracking was always performed in the unmown refuge zones, since butterflies stayed in these zones. If a tracked individual left the uncut zone, we continued to follow it up to 10 min.

### Statistical analyses

To describe the relative butterfly abundance in the unmown site, we fitted second-order polynomials to the densities observed in the transects at the different dates by least square differences for grassland butterflies, habitat generalist species and for *M*. *jurtina* males and females.

#### Mowing and relative abundance

Butterfly densities in the refuge zone before and after mowing were analysed by mixed models on ln (1 + number of individuals observed on a transect). Date (number of days since the beginning of the flight period of *M*. *jurtina*) and date^2^ were used as continuous, fixed factors. Butterfly group (grassland species vs. habitat generalist species), mowing stage (before or after mowing) and the interaction term were used as fixed factors. Site, year and the site x mowing interaction were introduced as random factors. For the analysis of *M*. *jurtina* densities, we applied the same model, with sex as a factor instead of butterfly group.

These analyses were performed with SAS 9.3 (mixed procedure) [36]. Covariance parameters were estimated by REML (residual maximum likelihood). Denominator degrees of freedom for the tests of fixed effects were estimated by the Satterthwaite method.

#### Behavioural changes observed during transect counts

A mixed binomial logistic model with a logit link (SAS glimmix procedure) was used to analyse the frequency of individuals observed feeding. Covariance parameters were estimated by RSPL (Random Solution Pseudo-Likelihood, corresponding to maximizing the residual log pseudo-likelihood with an expansion about the current solutions of the best linear unbiased predictors of the random effects; [36]). Denominator degrees of freedom for the tests of fixed effects were estimated by the Satterthwaite method. The binomial variable for each individual was “1”: feeding or “0”: not feeding (other activities pooled). Flower abundance was introduced as a covariate. The model contained mowing treatment, sex and the interaction term as fixed factors, while site, year and transect ID (nested within site) were included as random factors.

#### Flower head visitation rates

For the analysis of visitation rates on introduced flower heads, we performed a zero-inflated Poisson regression with mowing treatment, nectar plant species (*C*. *jacea* or *T*. *pratense*) and the interaction term as fixed factors, in *R* 2.15.1 [37] with packages pscl [38] and MASS [39].

#### Behavioural tracking: feeding and interacting

The proportion of time spent feeding vs. all other activities was analysed with a mixed logistic binomial model with sex, mowing treatment and the interaction effect as fixed factors, with SAS 9.3 software [36], using the same technique as for the behavioural changes observed during transect counts. Individual was included as a random factor. Next, we applied the same logistic binomial model to analyse proportion of nectaring events on *C*. *jacea*.

We counted the number of changes in activity per 10-min track and analysed it by negative binomial regression, with mowing, sex and their interaction effect as fixed factors. This was performed in *R* 2.15.1 [37] with packages pscl [38] and MASS [39].

Tracked females never interacted with other individuals. Hence, we analysed the number of interactions using a quasi GLM with Poisson distribution for males only, in *R* 2.15.1 [37] with packages pscl [38] and MASS [39]. Mowing was a fixed factor in the model. We analysed the duration of interactions using a mixed model for ln(duration) and with mowing as fixed factor and individual as random factor in SAS 9.3 [36].

#### Behavioural tracking: flight path analysis

A flight bout was defined as a whole flying event between two stops (either resting, basking or nectaring). We included only those flights that fulfilled at least one of three criteria: i) the flight lasted longer than 3 s; ii) it covered more than 30 cm; or iii) the activity before the flight differed from the activity after the flight. After mowing, we selected only the flight bouts that remained within the unmown zone; the mown meadow is a very different environment from the refuge and alters flight paths from typical foraging to straight dispersal flight [40]. For flight bouts, we analysed the i) flight distance (including the sinuosity of the flight track), and ii) net displacement (Euclidean distance between take-off and landing). We also analysed the duration, speed and linearity (i.e., the ratio between net displacement and real distance). Single flight distance, speed and flight duration were analysed using mixed models in SAS 9.3 [36], on log10-transformed data with sex, mowing and the interaction term as fixed effects and individual as a random effect. We used the same model for flight bout linearity after log10 (1-linearity) transformation to reach normality.

## Results

**Fig 1:**
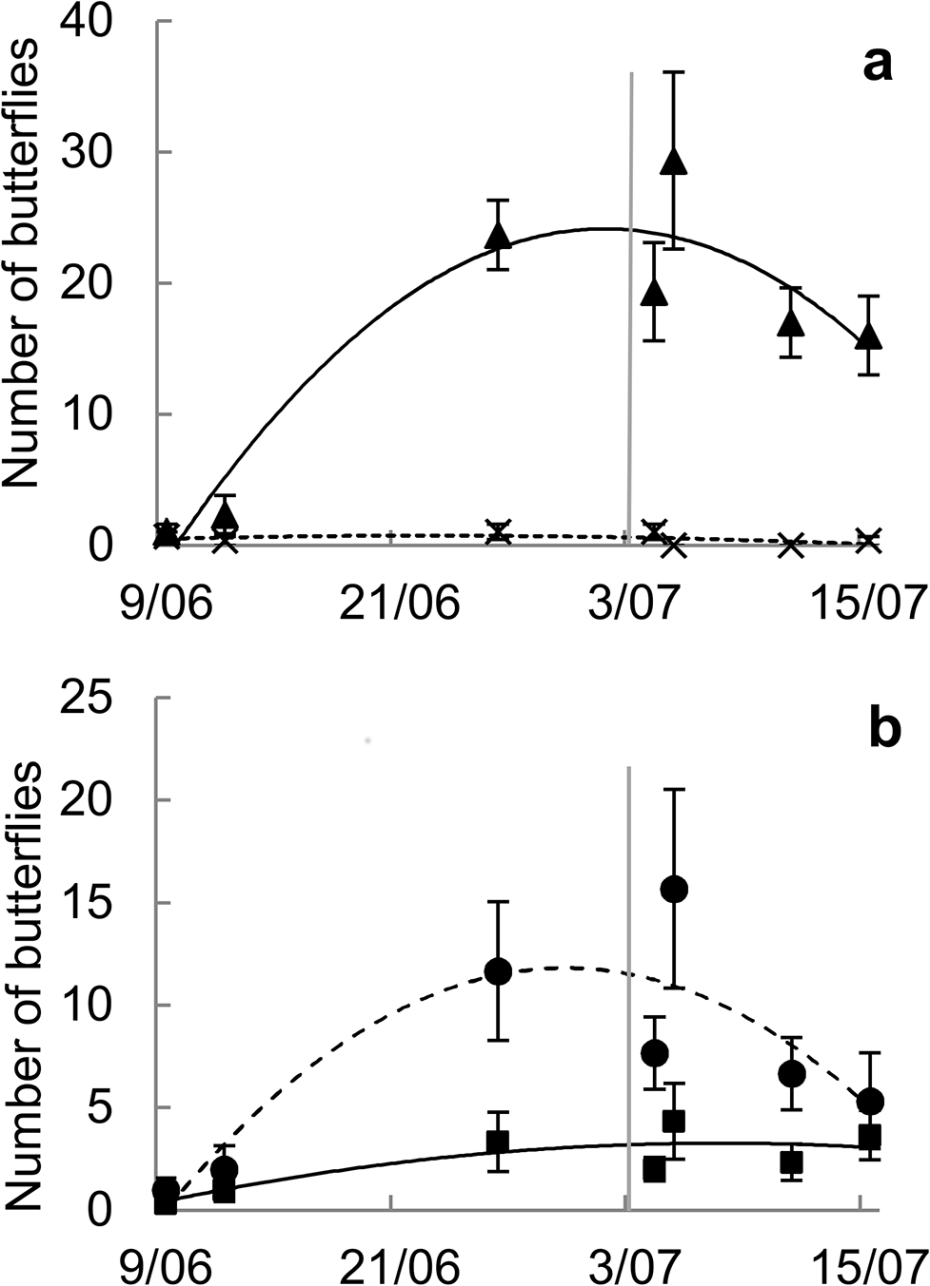
Butterfly phenology and mowing date in 2011. Mean number of butterflies (± SEM) recorded during transect counts in a site that was not mown, throughout the observation period in 2011. a) Grassland species (triangles, solid line) and generalist species (crosses, dotted line). b) *M*. *jurtina* females (circles, dotted line) and males (squares, solid line). Curves are second-order polynomial regressions fitted through least squares differences. Other studied sites in 2011 were mown on the 3^rd^ of July, indicated by the vertical line.

Grassland butterfly abundance increased from early June until the end of June, when it reached its maximum value, and then decreased until mid-July, whereas generalist butterflies remained relatively rare throughout the observation period (Fig 1A). Females of *M*. *jurtina* reached their peak abundance in 2011 at the end of June and decreased in abundance afterwards, while males were observed at lower densities throughout the observation period (Fig 1B).

### Mowing and butterfly abundance

**Fig 2:**
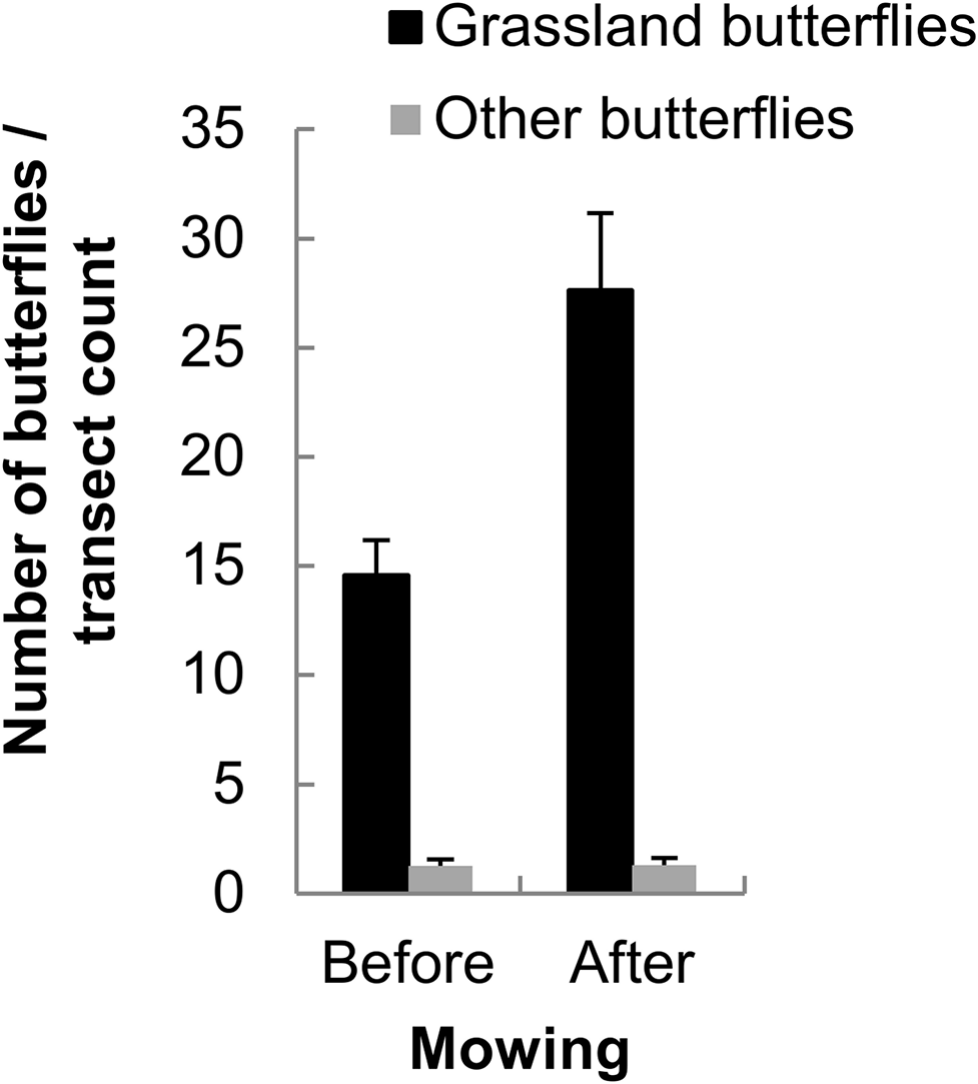
Increase in grassland butterflies abundance after mowing. Mean number of butterflies recorded during transect counts before and after mowing (± SE). Mixed model: Date: F1,152 = 6.82, P = 0.0099, Date^2^: F1,187 = 7.17, P = 0.0081, Mowing: F1,26.5 = 2.25, P = 0.145, Species group: F1,169 = 672.5, P < 0.0001, Mowing x Species group: F1,169 = 11.54, P = 0.0009. Ntotal = 2285 butterflies (809 before and 1476 after). Grassland species: *Maniola jurtina*, *Aphantopus hyperantus*, *Melanargia galathea*, *Coenonympha pamphilus*, *Pyronia tithonus*, *Papilio machaon*, *Thymelicus lineola*, *T*. *sylvestris*, *Thecla betulae*, *Heodes tityrus*, *Polyommatus icarus*, and *Lycaena phlaeas*. Other species (non grassland specialists): *Aglais urticae*, *A*. *io*, *Arashnia levana*, *Vanessa cardui*, *V*. *atalanta*, *Aporia crataegi* and pierid butterflies.

**Fig 3:**
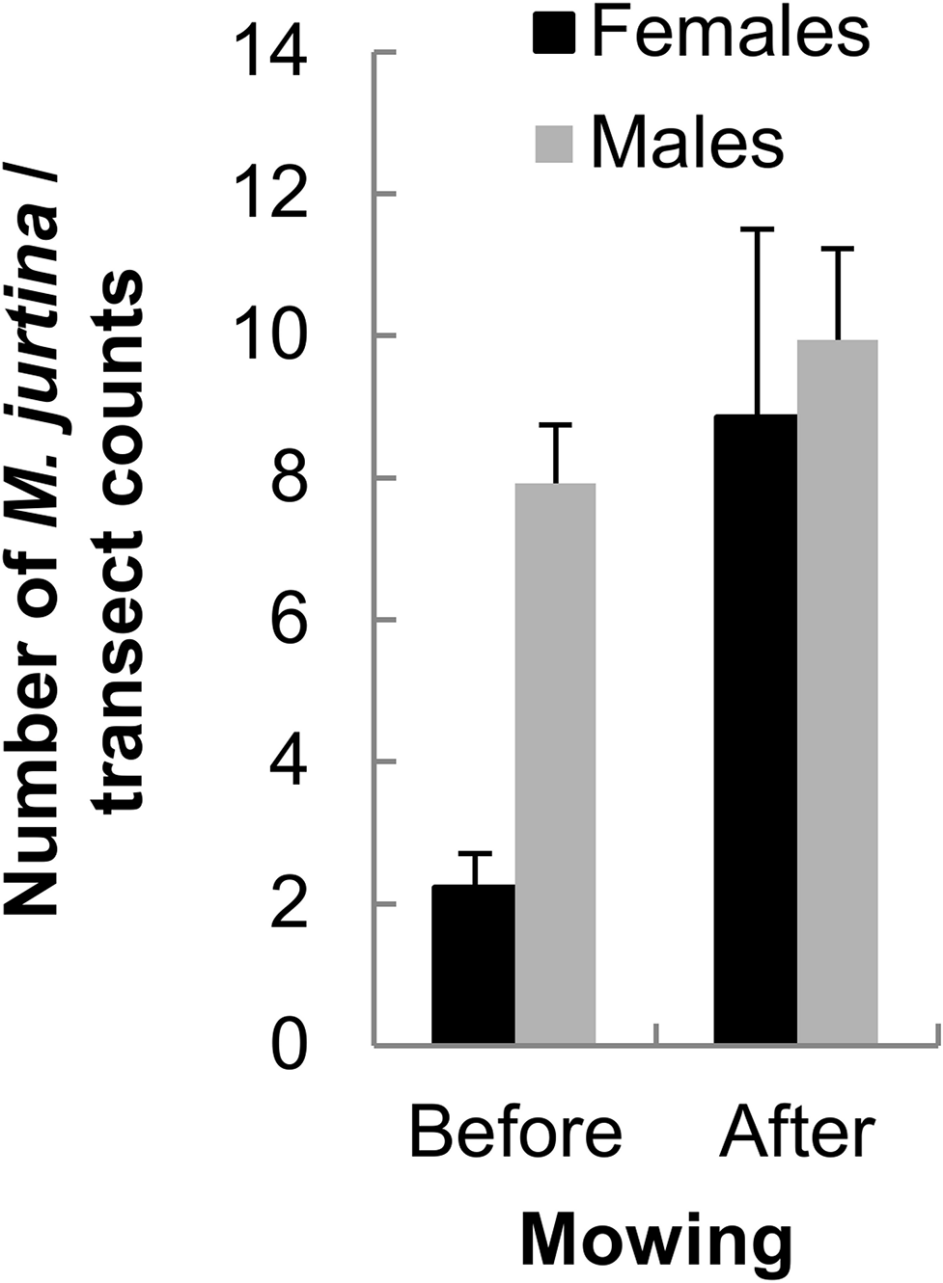
Increase in *M*. *jurtina* after mowing. Mean number (± SE) of *M*. *jurtina* recorded during transect counts in the refuge zone before and after mowing. Mixed model: Date: F_1, 180_ = 13.58, *P* = 0.0003, Date^2^: F_1, 196_ = 24.38, *P* < 0.0001, Mowing: F_1, 26.2_ = 6.04, *P* = 0.0209, Sex: F_1, 169_ = 65.7, *P* < 0.0001, Mowing x Sex: F_1, 169_ = 11.04, *P* = 0.0011. N_total_ = 1474 (562 females and 912 males).

After mowing, the abundance of grassland butterflies increased significantly in the refuge zone; it doubled on average, whereas there was no effect on generalist butterfly species (Fig 2). An increase in relative abundance was also observed in *M*. *jurtina*; the effect was modest in males, but female relative abundance increased on average fourfold after mowing (Fig 3). For the remainder of the results, we focus exclusively on *M*. *jurtina*.

### Feeding behaviour and nectar use

Flower visiting frequency was significantly influenced by flower abundance, butterfly sex and mowing. Higher frequencies of nectaring were observed in flower-rich sites and females showed higher frequencies than males (54.3% and 3.7%, respectively). The effect of mowing had a sex-specific effect, explaining the highly significant interaction effect (Table 1). Before mowing, 29.7% of the females were feeding, whereas this frequency increased to 60.3% after mowing. However, males did not feed more frequently after mowing (< 5% in both periods).

**Table 1:** Mixed binomial regression of feeding frequency of *M*. *jurtina* during transect counts relative to nectar supply, sex and mowing stage (before or after mowing).

Effect	DF	F	*P*
**Nectar offer**	24.7	4.83	0.0375
**Sex**	1469	124.05	<0.0001
**Mowing**	65.07	0.07	0.7911
**Sex x Mowing**	1469	6.95	0.0085

Camera observations on potted nectar plants in the field before and after mowing also showed an increase in feeding rate in the uncut strips after mowing (Table 2). Interestingly, the effect differed between nectar plant species (Table 2): it was very strong for the preferred *C*. *jacea* (mean number of visits h^-1^ ± SE: before 0.4 ± 0.4; after 5.0 ± 2.1), but absent for *T*. *pratense* (before 0.6 ± 0.4; after 0.25 ± 0.25).

**Table 2:** Zero-inflated Poisson regression of the number of visits by *M*. *jurtina* in 1-h video-recordings of visits to inflorescences of *Centaurea jacea* and *Trifolium pratense*.

Effect	Estimate	SE	z	*P*
**Intercept**	18.961	0.224	8.452	<0.0001
**Nectar plant species**	-29.450	11.086	-2.657	0.0079
**Mowing**	-23.686	0.913	-2.593	0.0095
**Nectar plant x Mowing**	32.699	15.313	2.135	0.0327

We tracked 109 individuals of *M*. *jurtina*, 68 females and 41 males, together they performed 400 nectaring visits: 162 (90 before mowing and 72 after) for females and 238 (118 before and 120 after mowing) for males, respectively. Behavioural data indicated that after mowing a larger part of their time was spent feeding (logistic binomial regression; Mowing: F_1, 88.03_ = 3.53, *P* = 0.064, Sex: F_1, 88.03_ = 2.59, *P* = 0.111, Mowing x Sex: F_1, 88.03_ = 1.05, *P* = 0.308); females spent 24.3% of their time to nectaring before mowing and 36.8% after, while males allocated respectively 23.3% and 44.8% of their time (Table 3).

**Table 3:** Mean duration in seconds (± SEM) of each activity as observed during 10-min behavioural trackings of *M*. *jurtina* females and males.

Activity	Females		Males	
	Before	After	Before	After
**Flying**	46 (8)	55 (10)	175 (27)	189 (25)
**Feeding on *C*. *jacea***	161 (33)	186 (47)	238 (42)	257 (26)
**Feeding on other species**	2 (2)	37 (20)	106 (99)	26 (8)
**Resting**	368 (37)	293 (45)	183 (27)	64 (17)
**Basking**	10 (8)	17 (9)	77 (21)	47 (10)
**Mating**	0	0	0	0
**Egg laying**	8 (5)	9 (3)	0	0
**Interacting with conspecifics**	3 (2)	2 (2)	21 (3)	32 (6)

After mowing, females visited other species than the preferred *C*. *jacea* more frequently (before: 1.1%, after: 29.5%; Table 3), while this was not the case for males (before: 13.5%, after: 7.5%) (logistic binomial regression: Mowing: F_1,115.4_ = 3.55, *P* = 0.062, Sex: F_1,115.4_ = 0.24, *P* = 0.626, Mowing x Sex: F_1,115.4_ = 5.5, *P* = 0.021). The ratio between the abundance of *C*. *jacea* and other flower species did not change between the two periods (22.6% before mowing and 23.5% after).

### Egg-laying behaviour

Before mowing, ovipositing females flew down into the grass to reach ground level, laid a single egg before flying away and repeating the sequence. Five females out of the 39 that were tracked were observed ovipositing and they laid a total of 16 eggs. After mowing, females almost always left the refuge zone and flew over variable distances in the mown zone before landing and ovipositing on a support or on bare ground in the mown zone. Then they flew further away to repeat the sequence before ultimately flying back to the refuge zone. Egg laying was the only activity observed in the mown zone after mowing. A total of 25 ovipositions performed by eight females (out of 29 tracked females) were observed, 21 of which were outside of the refuge zone.

### Flight characterization and interactions

We observed 235 interactions between *M*. *jurtina* males and other butterflies (148 before and 87 after mowing). Males did not interact more frequently with other butterflies after mowing (mean number of interactions during an observation session ± SE: 2.23 ± 0.42) than before (1.89 ± 0.31) (Quasi-GLM with Poisson distribution: Mowing: χ_1,115_ = 399.23, *P* > 0.53). However, intraspecific interactions lasted on average longer after mowing (5.2 ± 0.4 s and 3.9 ± 0.2 s, respectively; Mixed model: Mowing: F_1, 33.1_ = 5.69, *P* = 0.029).

Both sexes tended to switch between activities more frequently after mowing (mean number of switches ± SE: females: before: 12.2 ± 1.7, after: 18.8 ± 3.6; males: before: 25.7 ± 2.7, after: 41.4 ± 2.1) (Table 4).

**Table 4:** Negative binomial regression analysis of the number of activity changes by *M*. *jurtina* during behavioural tracks before and after mowing.

Variables ^a^	β	SE	*P*
**Sex**	0.789	0.271	0.0035
**Mowing**	-0.428	0.207	0.0386
**Sex x Mowing**	-0.047	0.341	0.889

^a^ Parameters estimates for sex give the value for males relative to females, and for mowing the values after mowing were used as reference level.

**Fig 4:**
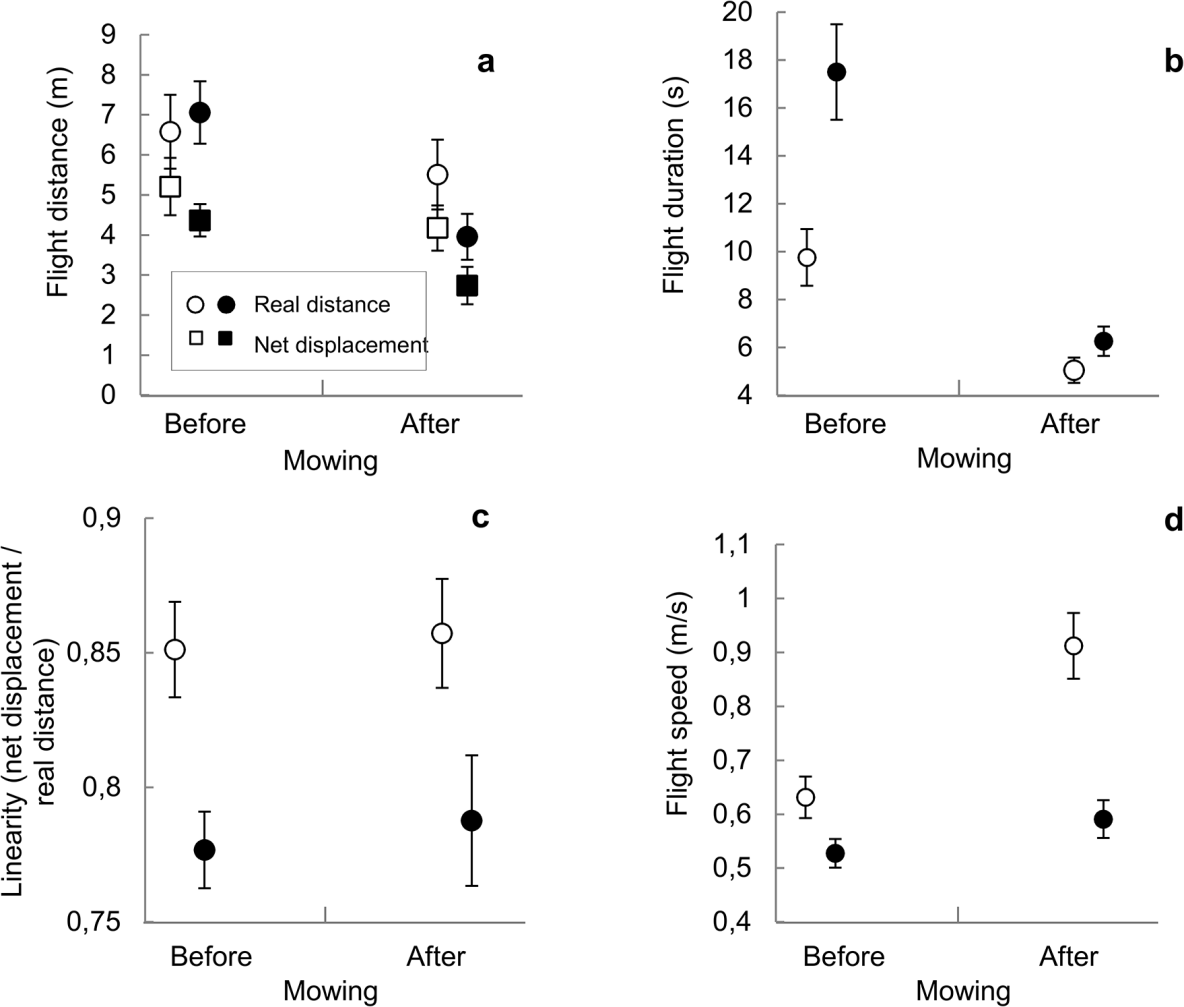
Flight traits of tracked *M*. *jurtina* before and after mowing. Open circles: females (n = 92 flight bouts before, 89 after); filled circles: males (n = 239 before, 111 after); N_total_ = 531. (a) mean flight distance, (b) mean flight duration, (c) mean linearity and (d) mean flight speed. Bars: ± 1 SE.

After mowing, single flight bouts of both males and females were significantly shorter; this was true for both total and net displacement. Flight bouts were also faster, but did not differ in linearity (Table 5, Fig 4). Males always tended to fly over shorter distances than females, but their flights took longer. Hence, males flew slower than females.

**Table 5:** The results of mixed models of flight behaviour variables of *M*. *jurtina* relative to the effect of mowing and sex.

Variable	Effect	df	F	*P*
**Flight duration**	Mowing	87.6	7.94	0.0060
	Sex	87.6	47.61	<0.0001
	Mowing x Sex	87.6	1.13	0.2901
**Flight distance**	Mowing	69.4	6.26	0.0147
	Sex	69.4	1.21	0.2749
	Mowing x Sex	69.4	0.23	0.6310
**Net displacement**	Mowing	67.8	7.89	0.0065
	Sex	67.8	3.19	0.0786
	Mowing x Sex	67.8	0.15	0.7028
**Linearity**	Mowing	75.8	1.94	0.1673
	Sex	75.8	0.45	0.5035
	Mowing x Sex	75.8	0.23	0.6294
**Speed**	Mowing	76.3	5.98	0.0168
	Sex	76.3	13.85	0.0004
	Mowing x Sex	76.3	0.81	0.3723

## Discussion

The basic challenge of mowing management from an animal conservation viewpoint is dealing with the compromise between short-term negative effects and long-term beneficial effects of keeping the vegetation in favorable condition, although other organisms may benefit from later successional stages of the vegetation [41]. The issue is highly relevant for species-rich meadows in nature reserves, but also for agri-environmental schemes in hay meadows and other grassy habitats placed on the edges of crop fields such as green lanes or conservation headlands. In hay meadows, sparing a zone from mowing in one year has often been recommended as a solution to control vegetation succession and avoid negative impacts on local animal populations (e.g. [10]). Although benefits are typically assumed [12,14], they are rarely demonstrated (but see [16,42]). Here, we provide quantitative evidence for a concentration effect after mowing in the refuge zone for flower-dependent insects, including grassland butterflies. Butterfly abundance and diversity were also high in linear grassy habitats on field edges implemented in agricultural landscape to promote biodiversity (e.g. [42, 43]), and are especially associated with nectar sources [44–46]. To avoid ecological succession, these linear grassy habitats are frequently managed by mowing, but timing of mowing relative to the butterfly’s flight season is known to play a major role for butterfly abundance [45,47]. For the meadow brown (*M*. *jurtina*), several of our results from behavioural observations point at higher levels of competition for resources in the refuge zone after mowing. These aspects have not been fully appreciated by conservation biologists and practitioners so far.

### Increased density of grassland butterflies and of *M*. *jurtina* in the refuge zone after mowing

The density of grassland butterflies in the refuge zone was almost doubled after mowing, whereas the density of habitat generalist butterflies remained stable. In 2011, mowing occurred after the butterfly abundance peak, hence the increased density in the refuge zone cannot be attributed to phenology. An increase in density in the refuge zone after mowing has recently been shown for grass-feeding insects (Orthoptera; [16]) and there is some evidence for displacements from mown to refuge zones in a few other arthropods [48,49]. The absence of a mowing effect on generalist butterfly species at the level of the local meadow most probably relates to their mobile life style and opportunistic use of nectar resources at a wider spatial scale across the landscape compared to typical grassland butterflies. Hence, the probability of encountering an individual of a generalist species in the refuge remained constant per unit area, resulting in the same densities before and after mowing.

For grassland butterflies like *M*. *jurtina*, higher densities after mowing are in line with the predicted concentration effect in the uncut refuge zone. At least in 2011, since peak abundance had already been reached before mowing, seasonal effects could be excluded as a cause for this increase. Some of the individuals could be immigrants from nearby meadows, but such an effect is likely to be more important if several meadows are mown simultaneously in a resource-poor landscape. However, Dover et al. [10] did not find evidence for increased dispersal of butterflies to nectar resources in adjacent meadows and tracks after mowing.

If we assume i) homogeneous habitat quality and butterfly abundance within a meadow and ii) that all grassland butterflies of a particular meadow would move to the uncut zone after mowing, then the increase in density should be inversely proportional to the part of the meadow left uncut. In our study, on average 10% of the meadows was left uncut, which would lead to the prediction of a tenfold increase in grassland butterfly density for the refuge zone. However, we observed on average only a doubling of the densities; for *M*. *jurtina* females we observed on average a fourfold increase. The discrepancy between the predicted and observed densities suggests significant losses for local populations, although with the current data we cannot discriminate between mortality and emigration. Direct killing due to hay cutting has been shown for several organisms [13], but rarely for butterflies at the adult stage, although its potential impact has been suggested by Dover et al. [10]. Moreover, butterflies from the surroundings could also immigrate to an unmown zone at any moment. In any case, meadows that are mown only once a year should be mown later in the season to guarantee floral resources to butterflies and other insects [50,51].

As our results suggest, unmown refuge zones offer opportunities for part of the local butterfly population after mowing of a hay meadow, but it appears to be far from a full compensation for the habitat loss induced by cutting. Long-term efficiency of a refuge zone in hay meadows has been shown for orthopterans, but they do not rely on nectar sources [42].

### Behavioural consequences of increased density for *Maniola jurtina*

Our study took the issue of unmown strips in meadows a step further by also addressing behavioural changes that are significant to butterfly survival and reproduction. Butterflies performed shorter and faster flight bouts after mowing and switched more frequently between different types of activities. This suggests a higher activity level in the refuge zone compared to the conditions before mowing. However, the frequency of intraspecific interactions did not increase and overall flight paths were similarly tortuous in the unmown zone before and after mowing. We confirmed sexual differences in several flight traits (*cf*. [30]), but they did not interact with the effect of mowing (Table 5). Increased male harassment could be a bigger issue if mowing occurred very early in the season, since *M*. *jurtina* is protandrous, but in our sample there was much variation in the timing of mowing (*see* Material and methods, Butterfly density and activity).

In our study areas, refuge zones were relatively narrow, linear elements (width: on average only 3 to 5 m) along the meadow border. It is known from studies on corridors that butterflies fly faster in narrow habitat elements [52]. As population density did not affect flight speed in another satyrine butterfly [53], we argue that the observed higher flight speed in the refuge zone after mowing is a result of a ‘canalising’ side effect of the geometry of the refuge. Little is known about the sensory ecology of flying butterflies under natural conditions, but visual cues are assumed to play an important role. There is an interesting scope for future work on sensory and cognitive issues of refuge use at the landscape scale.

The significantly higher frequency of switches between activities most likely relates to lower nectar rewards per flower visit, which in turn demanded higher frequencies of altering between foraging and flying. This likely implies an increase in the physiological cost, as insect flight is energetically costly and particularly so for take-offs [54]. Frequent switching between foraging and flight include frequent take-offs. Hence, the profitability of flower visits will be lower in the refuge after mowing.

Only a low proportion of the tracked females were observed ovipositing. However, our behavioural tracking was not specifically designed to study oviposition in any detail (unlike for example [55]). We argue that the 10-min track did not provide a sufficient time window as to reflect the proportion of egg-laying females in the population. Moreover, the majority of egg-laying occurs between 14.00 and 16.00 h and our behavioural trackings took place between 10.00 h to 17.00 h. The observation of females leaving the refuge zone after mowing to lay eggs reveals that they find resources (i.e., oviposition sites) within the mown zone. *M*. *jurtina* is known to emigrate from recently cut or grazed areas [30], and our observation are in agreement with former studies in that sense, since butterflies were absent from the mown and hence nectar-free zone of the meadows after mowing. However, the high proportion of oviposition episodes observed in the mown zone after mowing, and the fact that the mown zone is used only for egg laying, indicates a preference of females for this zone for oviposition, as previously observed [47]. Actually, the mown part of a meadow is different from the refuge in several aspects, including microclimate, predation risks and plant quality. Eggs laid in the mown zone have higher probabilities to successfully become adult butterflies than eggs laid in the refuge zone (Lebeau et al., unpublished data). Although the majority of our results showed negative effects of mowing for butterfly communities, we argue, based on these oviposition patterns, that it creates nevertheless new, beneficial conditions.

Our results showed an effect of mowing and of the consequent density increase for behaviour related to resource use. Butterflies–females in particular–allocated proportionally more time to foraging, as shown from our transect and individual tracking data. *Centaurea jacea* is a favourable and preferred nectar source in our sites and this nectar source was visited more often after mowing (based on camera data). This flower species is also frequently visited and highly appreciated by several bee species (honey bees, bumblebees, wild bees) [56], that will also be forced to forage within the remaining unmown zones after mowing or to move to other landscape elements. Nectar production in the *Centaurea* genus is fairly continuous throughout the day [57], so even in the case of total nectar depletion by previous visits, small amounts of nectar are likely to be available on inflorescences, although nectar secretion is relatively slow. However, wild *Centaurea* flowers in the field are always nearly empty, and nectar quantities within the florets were too small to be sampled with a microcapillary (Lebeau et al., unpublished data; [58]). Within the time spent feeding, a larger proportion of time was used to forage on less-preferred nectar species after mowing than before (based on individual track data). These results are best explained by higher levels of scramble competition for nectar at both the intra- and interspecific level after mowing. Quantity and quality of nectar affect butterfly fecundity, especially in females [59]. Experiments have established strong relationships between nectar use and butterfly fitness [60,61]. Female butterflies are typically more demanding for nectar (including carbohydrates and amino acids), because they have to manufacture eggs [62].

### Management practices favouring grassland butterfly community

Our results raise several questions about the optimal quantity and quality of uncut refuge zones in hay meadows for efficient conservation of grassland butterflies and other insects. The location, surface and nectar supply of a refuge zone are key factors in maximizing local resource availability and local survival after mowing. Agri-environmental schemes and management plans often focus on percentages, but the absolute area and shape of the refuge zone should also be considered. Although management techniques to stimulate nectar supply in the refuge zone deserve further attention, a local optimization of the nectar resources within a 5–10% strip of a meadow may not be sufficient for harbouring local populations of nectar-feeding insects. We did not study very small or large refuge zones, but based on our results one could still question the ‘10%-rule’ that is often applied for management regimes with a refuge zone. Our observations of oviposition in the mown zone after mowing suggest that the insect community would benefit from applying several cuts a year, on a fraction of the meadow only, as to finish the season with each fraction having been cut at least once, with uncut zones remaining throughout the year and varying in localization within the site. There is also scope for further work on the effects of local movements induced by mowing. Butterflies move to surrounding landscape elements to find more of the limiting resources (complementation), or other resources absent from the refuge (supplementation;[63]). Beside the quantity and spatial configuration of nectar resources, the qualitative aspects appear to be largely neglected. Butterflies have often been considered nectar-opportunistic, but different plant species may provide different nectar quantities and qualities with significant fitness consequences [61,64]. Further experiments on the area, configuration and quality of refuge zones are now warranted to provide evidence-based guidelines for hay meadow management.

### Conclusion

Sparing zones in hay meadows from cutting provides essential resources to grassland arthropods, including flower-visiting grassland butterflies. The principle is well-known and widely applied, but the consequences for local populations in the refuge zones have been largely ignored. We observed a significant increase in butterfly density in the refuge zone after mowing, but not enough to account for total population densities before mowing, indicating a local decline in butterfly abundance. We also showed evidence of changes in behaviour and flower visitation after mowing in line with increased scramble competition for (nectar) resources. Our study raises novel issues about the quantity, configuration and quality of refuge zones in grasslands for animal conservation.

## References

[1] Butler SJ, Vickery JA, Norris K (2007) Farmland biodiversity and the footprint of agriculture. Science (80-) 315: 381–384. 17234947 10.1126/science.1136607

[2] Robinson RA, Sutherland WJ (2002) Post-war changes in arable farming and biodiversity in Great Britain. J Appl Ecol 39: 157–176.

[3] Littlewood NA., Stewart AJA, Woodcock BA (2012) Science into practice—how can fundamental science contribute to better management of grasslands for invertebrates? Insect Conserv Divers 5: 1–8.

[4] Tscharntke T, Klein AM, Kruess A, Steffan-Dewenter I, Carsten T (2005) Landscape perspectives on agricultural intensification and biodiversity–ecosystem service management. Ecol Lett 8: 857–874.

[5] Öckinger E, Smith HG (2007) Semi-natural grasslands as population sources for pollinating insects in agricultural landscapes. J Appl Ecol 44: 50–59.

[6] Kohler F, Verhulst J, Van Klink R, Kleijn D (2008) At what spatial scale do high-quality habitats enhance the diversity of forbs and pollinators in intensively farmed landscapes? J Appl Ecol 45: 753–762.

[7] Tallowin JRB, Smith REN, Goodyear J, Vickery JA (2005) Spatial and structural uniformity of lowland agricultural grassland in England: a context for low biodiversity. Grass Forage Sci 60: 225–236.

[8] Humbert J-Y, Ghazoul J, Sauter GJ, Walter T (2010) Impact of different meadow mowing techniques on field invertebrates. J Appl Entomol 134: 592–599.

[9] Johst K, Drechsler M, Thomas J, Settele J (2006) Influence of mowing on the persistence of two endangered large blue butterfly species. J Appl Ecol 43: 333–342.

[10] Dover JW, Rescia A, Fungariño S, Fairburn J, Carey P, Lunt P et al (2010) Can hay harvesting detrimentally affect adult butterfly abundance? J Insect Conserv 14: 413–418.

[11] Vickery JA, Tallowin JR, Feber RE, Asteraki EJ, Atkinson PW, Fuller RJ et al (2001) The management of lowland neutral grasslands in Britain: effects of agricultural practices on birds and their food resources. J Appl Ecol 38: 647–664.

[12] Cizek O, Zamecnik J, Tropek R, Kocarek P, Konvicka M (2012) Diversification of mowing regime increases arthropods diversity in species-poor cultural hay meadows. J Insect Conserv 16: 215–226.

[13] Humbert J-Y, Ghazoul J, Walter T (2009) Meadow harvesting techniques and their impacts on field fauna. Agric Ecosyst Environ 130: 1–8.

[14] Braschler B, Marini L, Thommen GH, Baur B (2009) Effects of small-scale grassland fragmentation and frequent mowing on population density and species diversity of orthopterans: a long-term study. Ecol Entomol 34: 321–329.

[15] WallisDeVries MF, Poschlod P, Willems JH (2002) Challenges for the conservation of calcareous grasslands in northwestern Europe: integrating the requirements of flora and fauna. Biol Conserv 104: 265–273.

[16] Humbert J-Y, Ghazoul J, Richner N, Walter T (2012) Uncut grass refuges mitigate the impact of mechanical meadow harvesting on orthopterans. Biol Conserv 152: 96–101.

[17] Schultz CB, Dlugosch KM (1999) Nectar and hostplant scarcity limit populations of an endangered Oregon butterfly. Oecologia 119: 231–238. 28307973 10.1007/s004420050781

[18] Sang A, Teder T (2011) Dragonflies cause spatial and temporal heterogeneity in habitat quality for butterflies. Insect Conserv Divers 4: 257–264.

[19] Odendaal FJ, Turchin P, Stermitz FR (1989) Influence of host-plant density and male harassment on the distribution of female *Euphydryas anicia* (Nymphalidae). Oecologia 78: 283–288. 28312370 10.1007/BF00377167

[20] Delattre T, Vernon P, Burel F (2013) An agri-environmental scheme enhances butterfly dispersal in European agricultural landscapes. Agric Ecosyst Environ 166: 102–109.

[21] Aviron S, Herzog F, Klaus I, Schüpbach B, Jeanneret P (2011) Effects of wildflower strip quality, quantity, and connectivity on butterfly diversity in a Swiss arable landscape. Restor Ecol 19: 500–508.

[22] Carvell C, Meek WR, Pywell RF, Goulson D, Nowakowski M (2006) Comparing the efficacy of agri-environment schemes to enhance bumble bee abundance and diversity on arable field margins. J Appl Ecol 44: 29–40.

[23] Pywell RF, Warman EA, Sparks TH, Greatorex-Davies JN, Walker KJ, Meek WR et al (2004) Assessing habitat quality for butterflies on intensively managed arable farmland. Biol Conserv 118: 313–325.

[24] Blake RJ, Woodcock BA, Westbury DB, Sutton P, Potts SG (2010) New tools to boost butterfly habitat quality in existing grass buffer strips. J Insect Conserv 15: 221–232.

[25] Meek B, Loxton D, Sparks T, Pywell R, Pickett H, Nowakowski M (2002) The effect of arable field margin composition on invertebrate biodiversity. Biol Conserv 106: 259–271.

[26] Field RG, Gardiner T, Mason CF, Hill J (2006) Agri-environment schemes and butterflies: the utilisation of two metre arable field margins. Biodivers Conserv 16: 465–474.

[27] Aviron S, Jeanneret P, Schüpbach B, Herzog F (2007) Effects of agri-environmental measures, site and landscape conditions on butterfly diversity of Swiss grassland. Agric Ecosyst Environ 122: 295–304.

[28] Van Swaay C, Warren M, Loïs G (2006) Biotope Use and Trends of European Butterflies. J Insect Conserv 10: 189–209.

[29] Van Swaay CAM, Van Strien AJ, Harpke A, Fontaine B, Stefanescu C, Roy D et al (2013) The European Grassland Butterfly Indicator: 1990–2011. Luxembourg. 1–36 p.

[30] Brakefield PM (1982) Ecological studies on the butterfly *Maniola jurtina* in Britain. I. Adult behaviour, microdistribution and dispersal. J Anim Ecol 51: 713–726.

[31] Merckx T, Van Dyck H (2002) Interrelations among habitat use, behavior, and flight-related morphology in two cooccurring satyrine butterflies, *Maniola jurtina* and *Pyronia tithonus* . J Insect Behav 15: 541–561.

[32] Davies NB (1978) Territorial defence in the speckled wood butterfly (*Pararge aegeria*): The resident always wins. Anim Behav 26: 138–147.

[33] Schneider C, Dover J, Fry GLA (2003) Movement of two grassland butterflies in the same habitat network: the role of adult resources and size of the study area. Ecol Entomol 28: 219–227.

[34] Pollard E, Yates TJ (1993) Monitoring Butterflies for Ecology and Conservation London: Chapman and Hall.

[35] Cowley MJR, Thomas CD, Roy DB, Wilson RJ, León-Cortés JL, Gutiérrez D, et al (2001) Density–distribution relationships in British butterflies. I. The effect of mobility and spatial scale. J Anim Ecol 70: 410–425.

[36] SAS (2012) SAS/STAT 12.1 User’s Guide Cary NC: SAS Institute Inc.

[37] R Development Core Team (2013) R: A Language and Environment for Statistical Computing Vienna, Austria: R Foundation for Statistical Computing.

[38] Zeileis A, Kleiber C, Jackman S (2008) Regression Models for Count Data in R. J Stat Softw 27.

[39] Venables WN, Ripley BD (2002) Modern Applied Statistics with S 4th Editio Springer, editor New York.

[40] Delattre T, Burel F, Humeau A, Stevens VM, Vernon P, Baguette M (2010) Dispersal mood revealed by shifts from routine to direct flights in the meadow brown butterfly *Maniola jurtina* . Oikos 119: 1900–1908.

[41] Balmer O, Erhardt A (2000) Consequences of succession on extensively grazed grasslands for Central European butterfly communities: rethinking conservation practices. Conserv Biol 14: 746–757.

[42] Buri P, Arlettaz R, Humbert J-Y (2013) Delaying mowing and leaving uncut refuges boosts orthopterans in extensively managed meadows: Evidence drawn from field-scale experimentation. Agric Ecosyst Environ 181: 22–30.

[43] Croxton PJ, Hann JP, Greatorex-Davies JN, Sparks TH (2005) Linear hotspots? The floral and butterfly diversity of green lanes. Biol Conserv 121: 579–584.

[44] Dover JW (1997) Conservation headlands: Effects on butterfly distribution and behaviour. Agric Ecosyst Environ 63: 31–49.

[45] Feber RE, Smith HG, Macdonald DW (1996) The effects on butterfly abundance of the management of uncropped edges of arable fields. J Appl Ecol 33: 1191–1205.

[46] Dover JW (1996) Factors affecting the distribution of satyrid butterflies on arable farmland. J Appl Ecol 33: 723–734.

[47] Kulfan J, Štrbová E, Zach P (2012) Effect of vegetation and management on occurrence of larvae and adults of generalist *Maniola jurtina* L. (Lepidoptera) in meadow habitats. Polish J Ecol 60: 601–609.

[48] Hossain Z, Gurr GM, Wratten SD, Raman A (2002) Habitat manipulation in Lucerne *Medicago sativa*: arthropod population dynamics in harvested and “refuge” crop strips. J Appl Ecol 39: 445–454.

[49] Thorbek P, Bilde T (2004) Reduced numbers of generalist arthropod predators after crop management. J Appl Ecol 41: 526–538.

[50] Valtonen A, Saarinen K, Jantunen J (2006) Effect of different mowing regimes on butterflies and diurnal moths on road verges. Anim Biodivers Conserv 29: 133–148.

[51] Dahlström A, Lennartsson T, Wissman J, Frycklund I (2008) Biodiversity and traditional land use in South-Central Sweden: the significance of management timing. Environ Hist Camb 14: 385–403. doi: 10.3197/096734008X333572

[52] Pryke SR, Samways MJ (2001) Width of grassland linkages for the conservation of butterflies in South African afforested areas. Biol Conserv 101: 85–96.

[53] Wickman P-O (1988) Dynamics of mate-searching behaviour in a hilltopping butterfly, *Lasiommata megera* (L.): the effects of weather and male density. Zool J Linn Soc 93: 357–377.

[54] Berwaerts K, Van Dyck H (2004) Take-off performance under optimal and suboptimal thermal conditions in the butterfly *Pararge aegeria* . Oecologia 141: 536–545. 15309609 10.1007/s00442-004-1661-9

[55] Van Dyck H, Regniers S (2010) Egg spreading in the ant parasitic butterfly, *Maculinea alcon*: from individual behaviour to egg distribution pattern. Anim Behav 80: 621–627.

[56] Hirsch M, Pfaff S, Wolters V (2003) The influence of matrix type on flower visitors of *Centaurea jacea* L. Agric Ecosyst Environ 98: 331–337.

[57] Lack AJ (1982) Competition for pollinators in the ecology of *Centaurea scabiosa* L. and *Centaurea nigra* L. II. Observations on nectar production. New Phytol 91: 309–320.

[58] Lack AJ (1982) Competition for pollinators in the ecology of *Centaurea scabiosa* L. and *Centaurea nigra* L. III. Insect visits and the number of successful pollinations. New Phytol 91: 321–339.

[59] Boggs CL, Ross CL (1993) The effect of adult food limitation on life history traits in *Speyeria mormonia* (Lepidoptera: Nymphalidae). Ecology 74: 433–441.

[60] Mevi-Schütz J, Erhardt A (2005) Amino acids in nectar enhance butterfly fecundity: a long-awaited link. Am Nat 165: 411–419. 15791533 10.1086/429150

[61] Jervis MA, Boggs CL (2005) Linking nectar amino acids to fitness in female butterflies. Trends Ecol Evol 20: 585–587. 16701440 10.1016/j.tree.2005.08.015

[62] O’Brien DM, Boggs CL, Fogel ML (2004) Making eggs from nectar: the role of life history and dietary carbon turnover in butterfly reproductive resource allocation. Oikos 105: 279–291.

[63] Ouin A, Aviron S, Dover J, Burel F (2004) Complementation/supplementation of resources for butterflies in agricultural landscapes. Agric Ecosyst Environ 103: 473–479.

[64] Erhardt A, Rusterholz H (1998) Do Peacock butterflies (*Inachis io* L.) detect and prefer nectar amino acids and other nitrogenous compounds? Oecologia 117: 536–542. 28307679 10.1007/s004420050690

